#  Survey of Teen Noise Exposure and Efforts to Protect Hearing at School — United States, 2020

**DOI:** 10.15585/mmwr.mm6948a5

**Published:** 2020-12-04

**Authors:** John Eichwald, Franco Scinicariello

**Affiliations:** ^1^Office of Science, National Center for Environment Health, CDC; ^2^Office of Innovation and Analytics, Agency for Toxic Substances and Disease Registry, Atlanta, Georgia.

Noise-induced hearing loss (NIHL) is a substantial, often unrecognized, health problem. Various learning environments and activities in school settings are loud. Researchers have reported the prevalence of NIHL among U.S. adolescents ranging between 12.8% and 17.5%, suggesting that one in every six to eight middle and high school students (aged 12–19 years) has measurable hearing loss likely resulting from excessive noise exposure (*1*). Evidence suggests that even mild levels of hearing loss negatively affect auditory perception and cognitive skills.* CDC analyzed data from a sample of 817 youths aged 12–17 years who responded to the web-based YouthStyles survey in 2020. The survey measured the frequency of exposure to loud noise in school settings, the provision of hearing protection devices (HPDs) during exposure, and whether prevention techniques were part of their educational curriculum. Approximately three in four teenage students reported being exposed to loud sound at school, and nearly one half (46.5%) of respondents reported exposure to loud sounds at school on a regular basis. A majority of students (85.9%) reported that their school did not provide HPDs during classes or activities where they were exposed to loud sounds, and seven out of 10 reported they were never taught how to protect their hearing. Increasing youth’s awareness about the adverse health effects of excessive noise exposure and simple preventive measures to reduce risk can help prevent or reduce NIHL. Health care providers and educators have resources and tools available to prevent NIHL among school-aged children. Increased efforts are needed to promote prevention.

Schools in the United States utilize a variety of policies and practices to ensure that students and staff members are safe from a wide range of physical hazards, including excessive noise exposure. CDC reported that approximately one half of schools (56.5%) and school districts (61.3%) require students to use HPDs during classes or activities in which they are exposed to potentially unsafe noise levels (*2*). That study, a data source for a Healthy People 2020 objective (ECBP-4.6),^†^ demonstrated a marked decrease from 49.4% in 2006 to 35.0% in 2014 in the proportion of K-12 schools educating students in the prevention of vision and hearing loss.

The current study used data from Porter Novelli’s 2020 YouthStyles^§^ survey via Ipsos’ KnowledgePanel,^¶^ an online panel representative of the noninstitutionalized U.S. population. YouthStyles is part of a series of web-based surveys conducted to gather insights about U.S. consumers, including information about their health, attitudes, and behaviors. The survey was fielded during June 10–25, 2020; participants were youths aged 12–17 years residing with parents who are members of the adult SummerStyles panel. Members are randomly recruited by mail using probability-based sampling by address to reach respondents regardless of whether they have landline phones or Internet access. If needed, households are provided with a laptop or tablet and access to the Internet. Parents participated in their survey portion immediately before their child’s survey participation and provided electronic consent for their child to participate. Youth-adult dyad households who completed the survey received 10,000 cash-equivalent reward points (worth approximately $10) to be split between the parent and youth respondents. Respondents were not required to answer individual questions and could exit the survey at any time.

The resulting data were weighted to match March 2019 U.S. Census estimates. The adult data were weighted using nine factors: gender, age, household income, race/ethnicity, household size, education, census region, metro status, and parental status of children aged 12–17 years. Youth weights were based off the final adult weights (which incorporate the previously mentioned nine factors) and then adjusted for the following seven factors: youth gender, youth age, household income, youth race/ethnicity, number of teenagers in the household, census region, and metro status. Personal identifiers were not included in the data file. Three questions were included related to this study. Participants were asked to indicate their responses with a forced choice scale (e.g., always, usually, seldom, never). For analysis, researchers dichotomized student answers into two categories for student exposure (every day or two to four times per week/never or every few months), provision of HPDs (always or usually/seldom or never), and hearing protection education (never/at least once or several times). Analyses were conducted using SAS software (version 9.4; SAS Institute). The PROC SURVEYFREQ procedure of SAS was used for descriptive analysis. Multivariable logistic regressions were used to calculate adjusted odds ratios (aORs), 95% confidence intervals (CIs), and p-values (α = 0.05).

A total of 817 youths (among 1,700 sampled parents) qualified and completed the survey, for a response rate of 48.1%. Among the youths surveyed, 73.6% reported exposure to loud sound at school for >15 minutes a day and nearly one half (46.5%) reported exposure every day or two to four times per week (Table 1). Of those students who reported any exposure, the majority (85.9%) reported that their school did not provide HPDs (seldom or never) during classes or activities where they were exposed to loud sounds. In addition, 70.4% of all respondents reported that they never had a class or coursework that taught how to protect hearing from noise. Most surveyed students were White (51.6%) or lived in a metropolitan area (86.5%). There was no significant difference in exposure reported by sex (Table 2). Students in the South were more likely to be exposed to loud sounds at school on a regular basis than were students in the Northeast (aOR = 1.7; 95% CI = 1.1–2.5). Students at schools with classes or coursework providing information about hearing protection from noise were more likely to report that they were provided with hearing protection devices (aOR = 5.4; 95% CI = 3.3–8.9). Students from households with an average income ≥$150,000 were significantly less likely to have hearing protection provided by the school (aOR = 0.2; 95% CI = 0.1–0.5).

**TABLE 1:** Selected characteristics regarding youth’s exposure to loud sounds at school, the provision of hearing protection devices (HPDs) during classes or activities where they were exposed to loud sounds, and educational coursework on how to protect their hearing — Porter Novelli YouthStyles, United States, 2020

Characteristic	Unweighted no.	Weighted no.	All respondents weighted % (95% CI)
How often exposed to loud sounds at school*			
Every school day	170	179	22.0 (18.5–25.4)
Two to four times per week	197	221	24.5 (21.0–28.1)
Every few months	228	221	27.1 (23.5–30.7)
Never	218	215	26.4 (22.8–30.0)
**How often exposed to loud sounds at school (grouped)**			
Every day/Two to four times per week	367	379	46.5 (42.4–50.6)
Never/Every few months	446	435	53.5 (49.4–57.6)
All respondents	813	815	NA
**How often school provided HPD^†^**			
Always	20	19	3.2 (1.6–4.7)
Usually	61	66	11.0 (7.8–14.1)
Seldom	84	81	13.5 (10.4–16.6)
Never	430	434	72.4 (68.1–76.7)
**How often school provided HPD (grouped)**			
Always/Usually	81	85	14.1 (10.7–17.5)
Seldom/Never	514	515	85.9 (82.5–89.3)
**Hearing protection coursework^§^**			
Never	570	572	70.4 (66.6–74.1)
At least once	208	202	24.8 (21.4–28.3)
Several times	34	39	4.8 (2.8–6.8)
**Hearing protection coursework (grouped)**			
Never	570	572	70.4 (66.6–74.1)
At least once/Several times	242	241	29.6 (25.9–33.4)
**Sex**			
Male	410	417	51.1 (47.0–55.2)
Female	407	400	48.9 (44.8–53.0)
**Age group, yrs**			
12–14	417	401	49.0 (45.0–53.1)
15–17	400	416	51.0 (46.9–55.0)
**Race/Ethnicity^¶^**			
White	514	422	51.6 (47.5–55.8)
Black	63	110	13.4 (10.1–16.8)
Hispanic	136	201	24.6 (20.7–28.5)
Other/Multiracial	104	84	9.3 (8.1−12.6)
**Income, USD ($)**			
<50,000	179	231	28.3 (24.3–32.3)
50,000–84,999	194	198	24.2 (20.8–27.7)
85,000–149,999	277	245	30.0 (26.3–33.6)
≥150,000	167	143	17.6 (14.7–20.4)
U.S. Census region of residence**			
Northeast	148	131	16.0 (13.2–18.8)
Midwest	196	180	22.0 (18.8–25.2)
South	289	308	37.6 (33.6–41.7)
West	184	199	24.3 (20.7–27.9)
**Metropolitan statistical area status**			
Nonmetropolitan	119	110	13.5 (10.8–16.1)
Metropolitan	698	707	86.5 (83.9–89.2)

**Abbreviations:** CI = confidence interval; NA = not applicable.

* Panelists were asked “During a normal school year, how often were you exposed to loud sounds at school for more than 15 minutes a day, such as music or industrial arts classes, cafeteria, sporting or dance events? By loud sounds, we mean sounds so loud that you had to raise your voice to be heard by someone at arm’s length.”

^†^ Panelists were asked “How often does your school provide hearing protection devices, such as earplugs or earmuffs, during classes or activities where you are exposed to loud sounds, such as industrial arts classes and marching band?”

^§^ Panelists were asked “How often have you had a class or coursework that taught you about how to protect your hearing from noise?”

^¶^ Persons who identified as White, Black, Asian, or other or multiracial were all non-Hispanic. Persons who identified as Hispanic might be of any race.

** *Northeast:* Connecticut, Maine, Massachusetts, New Hampshire, New Jersey, New York, Pennsylvania, Rhode Island, and Vermont. *Midwest:* Illinois, Indiana, Iowa, Kansas, Michigan, Minnesota, Missouri, Nebraska, North Dakota, Ohio, South Dakota, and Wisconsin. *South:* Alabama, Arkansas, Delaware, District of Columbia, Florida, Georgia, Kentucky, Louisiana, Maryland, Mississippi, North Carolina, Oklahoma, South Carolina, Tennessee, Texas, Virginia, and West Virginia. *West:* Alaska, Arizona, California, Colorado, Hawaii, Idaho, Montana, Nevada, New Mexico, Oregon, Utah, Washington, and Wyoming.

**TABLE 2:** Multivariable logistic regression comparing frequencies of youths’ exposure to loud sounds at school, the provision of hearing protection devices (HPDs) during classes or activities where they were exposed to loud sounds, and educational coursework on how to protect their hearing — Porter Novelli YouthStyles, United States, 2020

Characteristic	aOR (95% CI)		
	Exposed to loud sounds at school every day/two to four times/week versus never/every few months*	School provided HPDs always/usually versus never/seldom^†^	Hearing protection coursework at least once/several times versus never^§^
**Sex**			
Male	Referent	Referent	Referent
Female	1.0 (0.8–1.4)	0.9 (0.6–1.5)	0.89 (0.7–1.2)
**Age group, yrs**			
12–14	Referent	Referent	Referent
15–17	0.9 (0.7–1.3)	1.2 (0.7–2.0)	1.11 (0.8–1.5)
**Race/Ethnicity^¶^**			
White	Referent	Referent	Referent
Black	0.7 (0.4–1.1)	1.7 (0.8–3.8)	1.4 (0.9–2.3)
Hispanic	1.1 (0.8–1.6)	1.3 (0.7–2.5)	1.3 (0.6–2.0)
Other/Multiracial	1.0 (0.6–1.5)	1.4 (0.6–3.4)	1.3 (0.7–2.2)
**Income, USD ($)**			
<50,000	Referent	Referent	Referent
50,000–84,999	1.2 (0.8–1.7)	0.6 (0.3–1.1)	1.4 (0.9–2.1)
85,000–149,999	0.8 (0.6–1.2)	0.9 (0.5–1.6)	0.9 (0.6–1.4)
≥150,000	0.8 (0.5–1.3)	0.2 (0.1–0.5)**	1.2 (0.7–1.9)
**U.S. Census region of residence^††^**			
Northeast	Referent	Referent	Referent
Midwest	1.3 (0.8–2.1)	0.8 (0.4–1.6)	0.8 (0.8–1.3)
South	1.7 (1.1–2.5)**	0.6 (0.3–1.2)	0.7 (0.4–1.1)
West	1.1 (0.7–1.7)	0.6 (0.3–1.4)	0.8 (0.5–1.3)
**Metropolitan statistical area status**			
Nonmetropolitan	Referent	Referent	Referent
Metropolitan	1.1 (0.7–1.7)	1.0 (0.5–2.0)	0.7 (0.4–1.0)
**How often taught to protect your hearing**			
Never	Referent	Referent	NA
Several/At least once	1.0 (0.8–1.4)	5.4 (3.3–8.9)**	NA
**How often exposed to loud sounds at school**			
Never/Every few months	NA	Referent	NA
Every day/two to four times per week	NA	1.2 (0.7–2.1)	NA

**Abbreviations:** aOR = adjusted odds ratio; CI = confidence interval; NA = not applicable.

* Panelists were asked “During a normal school year, how often were you exposed to loud sounds at school for more than 15 minutes a day, such as music or industrial arts classes, cafeteria, sporting or dance events? By loud sounds, we mean sounds so loud that you had to raise your voice to be heard by someone at arm’s length.”

^†^ Panelists were asked “How often does your school provide hearing protection devices, such as earplugs or earmuffs, during classes or activities where you are exposed to loud sounds, such as industrial arts classes and marching band?”

^§^ Panelists were asked “How often have you had a class or coursework that taught you about how to protect your hearing from noise?”

^¶^ Persons who identified as White, Black, Asian, or other or multiracial were all non-Hispanic. Persons who identified as Hispanic might be of any race.

** Statistical difference at p<0.05 compared with the referent group.

^††^ *Northeast:* Connecticut, Maine, Massachusetts, New Hampshire, New Jersey, New York, Pennsylvania, Rhode Island, and Vermont. *Midwest:* Illinois, Indiana, Iowa, Kansas, Michigan, Minnesota, Missouri, Nebraska, North Dakota, Ohio, South Dakota, and Wisconsin. *South:* Alabama, Arkansas, Delaware, District of Columbia, Florida, Georgia, Kentucky, Louisiana, Maryland, Mississippi, North Carolina, Oklahoma, South Carolina, Tennessee, Texas, Virginia, and West Virginia. *West:* Alaska, Arizona, California, Colorado, Hawaii, Idaho, Montana, Nevada, New Mexico, Oregon, Utah, Washington, and Wyoming.

## Discussion

This study suggests that approximately three in four students are exposed to loud sounds at school with nearly one half (46.5%) exposed on a routine basis. Among the students reporting exposure (73.6% of all respondents), 85.9% reported that they were not provided hearing protection during class or activities where they were exposed. Among all students responding, fewer than one out of three (29.6%) reported being taught how to protect their hearing during noisy events or activities. A loud sound level in a classroom is not just an annoyance; it can also disrupt academic performance and educational activities (*3*,*4*). Certain classroom environments as well as some related school activities can be loud and might contribute to NIHL (*5*,*6*). The finding that schools providing information about hearing protection were more likely to supply HPDs emphasizes the need for an increased public health focus on raising awareness about the adverse health effects of excessive noise exposure, as well as the importance of protective measures from both internal (e.g. classroom chatter or ventilation systems) and external background noises in school settings. Reported rates of HPD use might have been lowered because of limited use during participation in marching band; however, some school programs do offer special filtered musician earplugs for their students.

The findings in this report are subject to at least four limitations. First, data are subject to sampling biases because data could be collected only from youths who chose to respond to the survey and had participating parents who provided consent for them to participate. Second, the data obtained in this survey were self-reported, relying on respondents’ perception of loudness and recall of events. Third, the survey did not ask parents whether their child attended a public or private school. Finally, there were small numbers in certain groups, resulting in wide CIs for estimates for these subgroups.

Both the World Health Organization (*7*) and the U.S. National Academies of Sciences, Engineering, and Medicine (*8*) have recommended that national governments improve public information on hearing and hearing health care through educational awareness campaigns. Promotion of three simple prevention techniques can protect hearing from excessive noise exposure: lowering the volume of audio equipment and devices, moving away from the sound source, and wearing hearing protectors, such as earplugs or earmuffs.

The Noisy Planet campaign** developed by the National Institutes of Health and the public health educational materials developed by CDC’s NIHL program^††^ in the National Center for Environmental Health (NCEH) are designed to increase awareness of the negative health effects from loud noise exposure. The Dangerous Decibels program^§§^ has developed effective classroom-based educational materials on hearing loss prevention designed to increase knowledge and positively change attitudes and intended behaviors of school-aged children (*9*).

NCEH has created educational products targeted specifically for school-aged children, including a downloadable 10-page graphic novel, How Loud is Too Loud?^¶¶^ In an agreement with Scholastic Magazine, 11,871 hard copies were distributed with the April 2020 edition of their SuperScience magazine to teachers of grades 3–6 in all 50 states, the District of Columbia, Guam, and the Armed Forces Europe/Armed Forces Pacific schools. A four-page standards-based teacher’s guide with lesson plans was included with the comic. Both tools provide information about NIHL and promote the three prevention techniques. Discussions between patients and health care providers regarding the consequences of excessive sound exposure and the potential benefits to health from the use of hearing protection might provide opportunities to prevent or reduce harmful effects. Educators, as well as school audiologists and nurses, have free resources and tools available to teach youths about the causes and prevention of NIHL.

**Summary**

**What is already known about this topic?**

Noise-induced hearing loss is a substantial, often unrecognized, health problem. Various learning environments and activities in school settings are loud.

**What is added by this report?**

Approximately three in four teenage students report being exposed to loud sound at school, and nearly one half (46.5%) report exposure on a regular basis. However, most report that their school did not provide hearing protection equipment or teach preventive techniques to reduce their risk of permanent noise-induced hearing loss.

**What are the implications for public health practice?**

Health care providers and educators have resources available to prevent noise-induced hearing loss among school-aged children. Increasing youths’ awareness about adverse health effects of excessive noise exposure and simple preventive measures to reduce risk can help prevent or reduce noise-induced hearing loss.

## Footnotes

* https://www.biorxiv.org/content/10.1101/723635v1.

† https://www.healthypeople.gov/node/4255/data_details.

§ http://styles.porternovelli.com/consumer-youthstyles/.

¶ https://www.ipsos.com/en-us/solutions/public-affairs/knowledgepanel.

** https://www.noisyplanet.nidcd.nih.gov.

†† https://www.cdc.gov/nceh/hearing_loss.

§§ http://dangerousdecibels.org.

¶¶ https://www.cdc.gov/healthyschools/bam/pdf/how_loud_is_too_loud-508.pdf.
